# Non-Coding RNAs in Hereditary Kidney Disorders

**DOI:** 10.3390/ijms22063014

**Published:** 2021-03-16

**Authors:** Julie Xia Zhou, Xiaogang Li

**Affiliations:** 1 Department of Internal Medicine, Advent Health, Orlando, FL 32804, USA; zhouxia1214@gmail.com; 2 Department of Internal Medicine, Mayo Clinic, Rochester, MN 55905, USA; 3 Department of Biochemistry and Molecular Biology, Mayo Clinic, Rochester, MN 55905, USA

**Keywords:** non-coding RNA, microRNA, Genetic kidney disease, PKD

## Abstract

Single-gene defects have been revealed to be the etiologies of many kidney diseases with the recent advances in molecular genetics. Autosomal dominant polycystic kidney disease (ADPKD), as one of the most common inherited kidney diseases, is caused by mutations of PKD1 or PKD2 gene. Due to the complexity of pathophysiology of cyst formation and progression, limited therapeutic options are available. The roles of noncoding RNAs in development and disease have gained widespread attention in recent years. In particular, microRNAs in promoting PKD progression have been highlighted. The dysregulated microRNAs modulate cyst growth through suppressing the expression of PKD genes and regulating cystic renal epithelial cell proliferation, mitochondrial metabolism, apoptosis and autophagy. The antagonists of microRNAs have emerged as potential therapeutic drugs for the treatment of ADPKD. In addition, studies have also focused on microRNAs as potential biomarkers for ADPKD and other common hereditary kidney diseases, including HNF1β-associated kidney disease, Alport syndrome, congenital abnormalities of the kidney and urinary tract (CAKUT), von Hippel–Lindau (VHL) disease, and Fabry disease. This review assembles the current understanding of the non-coding RNAs, including microRNAs and long noncoding RNAs, in polycystic kidney disease and these common monogenic kidney diseases.

## 1. Introduction

For many decades, it was initially thought that the majority of transcriptomes are mRNAs, which are able to translate into proteins based on the code in the mRNAs. However, only approximately 2% of human genes were identified to encode proteins, and the majority of human genes are transcribed into noncoding RNAs (ncRNAs) from the recent findings of the Encyclopedia of DNA Elements (ENCODE) Project Consortium [1]. The ENCODE project also provided the evidence that noncoding genes could be pervasively transcribed to regulate protein-coding genes via forming complex regulatory networks [1]. Thus, understanding the role of ncRNAs in human diseases has become one of the most important challenges of science.

**Figure 1:**
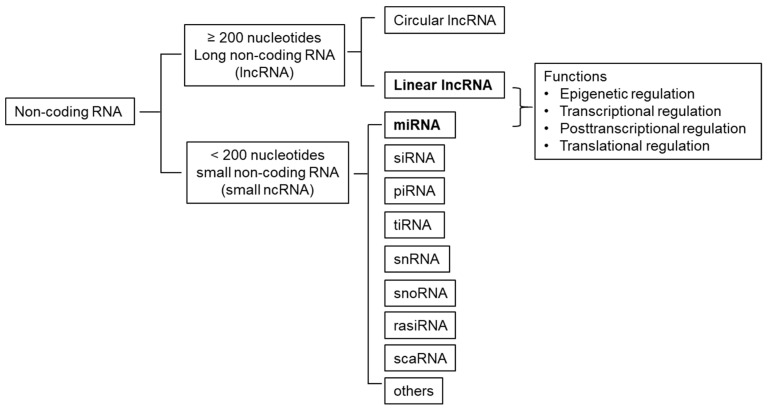
Classifications of non-coding RNA (ncRNA), including long non-coding RNA (circular and linear lncRNA) and small non-coding RNA (microRNA (miRNA), small interfering RNA (siRNA), piwi-interacting RNA (piRNA), transfer RNA-derived stress-induced small siRNA (tiRNA), small non-coding RNA (snRNA), small nucelolar RNA (snoRNA), small cajal body-specific RNA (scaRNA), and others). The functions of lncRNA and miRNA can be epigenetically, transcriptionally, and post-transcriptionally regulated.

ncRNAs are mainly classified into long ncRNAs (lncRNA s) and small ncRNAs based on their length, using a cutoff of 200 nucleotides (Figure 1) [2]. LncRNAs are longer than 200 nucleotides, which comprise linear lncRNAs (named by default as lncRNAs) and circular RNAs (circRNAs). Small ncRNAs are shorter than 200 nucleotides, which include microRNA (miRNA), small interfering RNA (siRNA), piwi-interacting RNA (piRNA), transfer RNA-derived stress-induced small siRNA (tiRNA), small nuclear ribonucleic acid (snRNA), small nucelolar RNA (snoRNA), repeat-associated small interfering RNA (rasiRNA), small cajal body-specific RNA (scaRNA) and others. Among them, miRNAs are the most extensively investigated small ncRNAs, in which the first miRNA lin-4 was identified in Caenorhabditis elegans in 1993 [3]. MiRNAs play a critical role in development and pathogenesis of a variety of diseases, such as cancer, diabetes, cardiovascular disease, and kidney diseases [4]. The dysregulated miRNAs are potential therapeutic targets of human diseases [5]. Given that the miRNAs in body fluids are stable and easily detectable, and have tissue-enriched expression profile, they have been reported to be used as potential diagnostic and prognostic biomarkers [5].

Inherited kidney diseases, consisting of monogenic and polygenic kidney disorders, have significant risk for the development of end-stage renal disease (ESRD). Monogenic kidney disease results from a pathogenic mutation of a single causative gene (Table 1), and more monogenic nephropathy genes are continually being identified by whole exome sequencing and whole genome sequencing [6]. Approximately 450 monogenic kidney disorders accounts for 30% cases of chronic kidney disease (CKD) in pediatric cohorts and 5–30% in adult cohorts [6]. Autosomal dominant polycystic kidney disease (ADPKD) is caused by mutations in PKD1 or PKD2 gene and is the most common monogenic kidney disorder [7]. The cyst-lining epithelial cells of PKD are hyperproliferative and hypersecretory, which leads to progressive cyst growth and expansion, and ultimately causes ESRD [7]. Recent evidence suggests that, in addition to the underlying gene mutation, epigenetic regulators modulate cyst growth and act as potential therapeutic targets [8]. One example is the role of miRNA-mediated signaling in promoting cyst formation [9]. This review focuses on the recent advances in understanding of the role of ncRNAs in the pathogenesis of PKD, discuss the potential application of ncRNA as therapeutic targets of PKD. In addition, this review also summarizes the findings of ncRNAs in other common monogenic kidney diseases, including HNF1β-associated kidney disease, Alport syndrome, congenital abnormalities of the kidney and urinary tract (CAKUT), von Hippel–Lindau (VHL) disease, and Fabry disease.

## 2. miRNAs

miRNAs, the 20- to 22-nucleotide-long RNA molecules, mainly downregulate gene expression post-transcriptionally [10]. Most miRNAs are processed by canonical miRNA biogenesis pathway [11]. Primary miRNAs (pri-miRNAs) are transcribed from their genes by RNA polymerase II enzyme, which are then cleaved into pre-miRNAs by the microprocessor complex that consists of an RNA binding protein DiGeorge Syndrome Critical Region 8 (DGCR8) and a ribonuclease III, DROSHA [12]. The pre-miRNAs are exported out of nucleus by an exportin 5/RanGTP complex [12]. The cytoplasmic pre-miRNAs are processed by the RNase III endonuclease Dicer, resulting in the formation of 22-nucleotide double stranded mature miRNAs [13]. The mature miRNA duplex is loaded into the Argonaute (AGO) family of proteins in an ATP-dependent manner, to form the miRNA-induced silencing complex (miRISC) [14]. AGO selects a “guide” strand based in part on the thermodynamic stability at the 5′ ends of the miRNA duplex [15]. The guide strand has a lower 5′ stability that is preferentially loaded into AGO [15]. The unloaded strand becomes the passenger strand that is cleaved by AGO2 and degraded by cellular machinery [15]. Multiple non-canonical miRNA biogenesis pathways, including Drosha/DGCR8-independent and Dicer-independent pathways, have been elucidated. In Drosha/DGCR8-independent pathway, the pre-miRNAs resemble Dicer substrates without the cleavage of Drosha [11]. On the other hand, in the Dicer-independent pathway, miRNAs are processed by Drosha, and can be loaded into AGO without the cleavage of Dicer [11].

The miRISC interacts with 3′ UTR of target mRNAs via miRNA response elements (MRE), MREa complementary sequence that is 2–8 nucleotides located at 5′ end of miRNAs. It has been reported that miRNAs also interact with other regions, including 5′ UTR, coding sequence, and gene promoter [16]. The binding of miRNA with MRE at 3′ UTR of target mRNAs results in mRNA deadenylation and decapping, mRNA cleavage by activation of AGO2 endonuclease, and translation repression [17,18,19]. The binding of miRNAs to 5′ UTR and coding regions of mRNAs downregulates the gene expression [20,21], whereas the binding of miRNAs to the promoter region has been reported to induce transcription [22].

It has been reported that miRNAs play critical roles in kidney development, maintaining homeostasis, acute kidney injury (AKI), and the progression of tubulointerstitial fibrosis [9,23,24]. Deletion of Dicer mediated by Six2-Cre in the progenitors of the nephron epithelium induces apoptosis and premature termination of nephrogenesis [25], in which deletion of Dicer in nephron progenitors induces apoptosis during kidney development that is regulated through increasing the expression of pro-apoptotic protein Bim [26]. Bim is targeted by several miRNAs, including miR-10a, miR-106b, and miR-17-5p, in nephron progenitors [26]. Loss of Dicer mediated by HoxB7-Cre in the ureteric bud epithelium also increases cell proliferation and apoptosis, and disrupts ciliogenesis, which leads to development of cysts [25]. As miRNAs are widely involved in the pathogenesis of AKI and CKD, they should have promising diagnostic and therapeutic potential [27,28].

## 3. LncRNAs

Unlike the extensive investigation of miRNAs over the past decade, the information regarding the function of lncRNAs is limited. lncRNAs are categorized as sense, antisense, intronic, intergenic, bidirectional and enhancer-associated on the basis of location with respect to protein-coding genes [29]. Growing evidence suggests that lncRNAs are central players in epigenetic regulation of tissue homeostasis during development and disease [30,31]. lncRNAs are enriched in the nucleus and associated with chromatin remodeling complex, thereby regulating the chromatin architecture of genes either in *cis* (near their transcription sites), or in *trans* (at sites distant from their transcription site) [32]. lncRNAs also regulate the recruitment of chromatin modifiers and transcription via a variety of mechanisms [32]. IncRNAs can also be exported to cytoplasm to regulate mRNA stability, modulate translation and interfere with posttranslational modifications [32]. More studies in the past five years have focused on the functional role of lncRNAs in kidney diseases, such as glomerular diseases, tubulointerstitial disease, kidney fibrosis, and acute kidney injury.

## 4. Noncoding RNA in Polycystic Kidney Disease

### 4.1. miRNAs and lncRNAs in ADPKD

**Figure 2:**
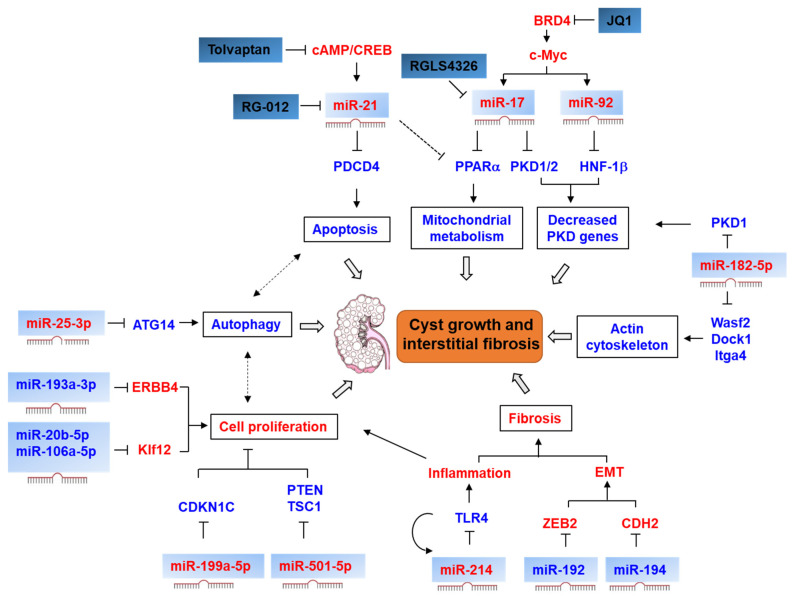
Signaling pathways modulated by miRNAs in autosomal dominant polycystic kidney disease (ADPKD). In ADPKD, miR-21 is upregulated by cAMP/CREB signaling, which represses the expression of PDCD4 to inhibit cystic renal epithelial cell apoptosis. The expression of miR-17 and miR-19 is transcriptionally activated by c-Myc, and the upregulation of miR-17 results in a defect of mitochondrial metabolism by repressing proliferator-activated receptor-α (PPARα). Furthermore, miR-17 represses the expression of *PKD1* and *PKD2*, and miR-92 represses the expression of *HNF-1β*. The upregulation of miR-25-3p inhibits autophagy by targeting ATG14. The downregulation of miR-193a-3p promotes cell proliferation through derepressing the expression of ERBB4. The downregulation of miR-20b-5p and miR-106-5p increases cell proliferation through derepressing the expression of Kif12. The upregulation of miR-199a-5p and miR-501-5p promotes cell proliferation through targeting CDK1N1C, PTE and TSC1, respectively. The upregulation of miR-214 inhibits inflammation through targeting TLR4. The downregulation of miR-192 and miR-194 promotes epithelial–mesenchymal transition (EMT) through targeting ZEB2 and CDH2. The upregulation of miR-182-5p decreases the expression of *PKD1* and leads to defects of actin cytoskeleton through targeting Wasf2, Dock1 and Itga4. Tovalptan, a vasopressin receptor antagonist. JQ1, a BRD4 inhibitor. RG-012, an anti-miR targeting miR-21. RGLS4326, an anti-miR target miR-17. The upregulation or activation of miRNAs and signaling pathways in ADPKD is marked in *red*. The downregulation or inhibition of signaling pathways in ADPKD is marked in *blue*. Arrows indicate a positive effect. “T” indicates a negative effect. Dashed lines indicate putative signaling pathways and mechanisms.

Numerous miRNAs have been investigated in PKD cells and murine models, and human ADPKD. The dysregulated miRNAs modulate cyst growth and interstitial fibrosis through a variety of mechanisms, including directly repressing the expression of PKD genes, regulating cystic cell proliferation, apoptosis, and autophagy, promoting epithelial–mesenchymal transition (EMT) and inflammation, and causing defects in mitochondrial metabolism and actin cytoskeleton (Figure 2). In the following sections, we discuss the dysregulation of different miRNAs in ADPKD (Table 2).

#### 4.1.1. mIR-17–92 Cluster

miR-17–92 cluster is an evolutionarily conserved oncogenic miRNA cluster, which encodes six miRNAs (miR-17, miR-18a, miR-19a, miR-19b-1, miR20a, and miR-92a-1). Deletion of a region of the chromosome 13 that includes MIR17HG, encoding human miR-17–92 cluster, causes type 2 Feingold syndrome [49]. Type 2 Feingold syndrome is autosomal dominant, and is characterized by abnormalities of fingers and toes, hearing loss, short stature, or kidney or heart abnormalities [49]. Mice with germline deletion of miR-17–92 are perinatal lethal with lung hypoplasia and a ventricular septal defect, and B cell maturation defect [50]. Conditional knockout of miR17–92 in nephron progenitors reduces the number of developing nephrons, which leads to albuminuria, podocyte foot process effacement and glomerulosclerosis in adult mice [51]. However, inducible deletion of miR-17–92 in adult mice leads to no obvious abnormalities [52], and kidney-specific knockout of miR-17–92 does not cause any changes of kidney morphology and histology [33].

It has been reported that miR17–92 cluster is upregulated in kidneys of multiple orthologous models of PKD and human ADPKD via c-Myc which directly binds to the conserved Myc binding sites on the promoter of miR17–92 cluster [33,34]. The oncogene c-Myc has been found to be upregulated in PKD and to promote cyst progression, and c-Myc transgenic mice develops renal cysts [53,54]. Inhibition of c-Myc through targeting its upstream epigenetic regulator BRD4 via bromodomain inhibitor JQ1 slowed cyst growth in PKD mouse models [54]. Similar to c-Myc transgenic mice, the kidney-specific transgenic overexpression of miR-17–92 develops renal cysts. Conditional knockout of miR-17–92 in PKD models slows cyst growth, preserves renal function, and prolongs the survival of those mice via inhibiting cell proliferation. Furthermore, anti-miR-17 attenuates cyst growth in two PKD animal models, and reduces cyst growth in in vitro models of human ADPKD. Bioinformatic analysis revealed that the 3′ UTR of PKD1 and PKD2 mRNA contains conserved binding sites for miR-17, and 3′ UTR of HNF-1b mRNA contains a miR-92 binding site [33,55]. The transcription factor hepatocyte nuclear factor-1β (HNF-1β) is encoded by HNF-1b, which directly regulates the transcription of PKD2 and PKHD1 [56]. The upregulated miR-17–92 negatively regulates the expression of PKD genes (PKD1, PKD2, and HNF-1b) in a posttranscriptional manner [33]. The disease severity of PKD is associated with the functional PC1 dosage which is suggested from the findings in hypomorphic PKD1 p.R3277C mouse model [57]. Thus, the potential mechanism by which miR17–92 promotes cyst growth may be through decreasing the expression of PKD genes.

A recent study has further identified that miR-17 is the primary pathogenic miRNA to promote cyst growth within miR17–92 family through in vivo screening of anti-miRNAs targeting miR-17, miR-18, miR-19 or miR-25 individually [35]. Anti-miR-17 treatment slowed cyst growth in *Pkd1^flox/RC^:Ksp-Cre* mice, a mouse model that carries a flox allele and a R3277C mutant allele of *Pkd1* gene, through regulating mitochondrial metabolism, mTOR pathway, and inflammation [35]. Specifically, miR-17 inhibits the expression of peroxisome proliferator-activated receptor-α (PPARα) by binding to the 3′-UTR of its mRNA. miR-17 downregulated the expression of PPARα target genes in cystic kidneys, including Pparg, Ppargc1a, Sod2, Me, Oxct1, Pdk4, Etfa, Etfb, Etfdh, Cd36, Slc27a2, and Cpt2. PPARα is the key regulator of mitochondrial oxidative phosphorylation (OXPHOS) and fatty acid oxidation (FAO), suggesting that miR-17 promotes cyst growth through affecting the mitochondrial metabolism in renal epithelial cells. These findings also indicate that miR-17-PPARα axis-mediated mitochondrial dysfunction is one of the alterations leading to the pro-proliferative metabolic reprogramming of cyst epithelia, in addition to the defective glucose metabolism and dysregulated lipid and amino acid metabolism [58,59,60]. Lee et al. has identified RGLS4326 by screening a chemically diverse library of anti-miR-17 oligonucleotides [61]. RGLS4326 is a single-stranded, chemically modified, short oligonucleotide with nine nucleotides that is complementary to the miR-17 seed sequence [61]. The safety of RGLS4326 is supported by the fact that no hematopoietic and renal toxicity are observed in monkeys [61]. RGLS4326 shows preferential kidney distribution, and mainly presents in both proximal tubules and collecting ducts in cystic kidneys of ADPKD mouse model [61]. Treatment with RGLS4326 suppresses cyst growth in human ADPKD models in vitro, and slows cyst growth in *Pkd2* conditional knockout mice (*Pkd2^flox/flox^:Pkhd1-Cre*) as well as *Pcy/CD1* and *Pcy/DBA* mice which develop polycystic kidney disease with mutations in NPHP3 (nephronophthisis 3) and are used as mouse models for long-term treatment [61]. By displacing miR-17 from translationally active polysome fractions, RGLS4326 de-represses the expression of miR-17 target genes, including *PKD1* and *PKD2* [61]. RGLS4326 treatment also normalizes the dysregulated metabolism pathways and inhibits the pro-proliferative pathways in cystic kidneys [61]. RGLS4326 is a potential drug candidate for ADPKD, due to its safety, stability, and therapeutic efficacy in PKD models. A Phase 1b clinical trial is designed to evaluate the safety, tolerability, pharmacokinetics, and pharmacodynamics of RGLS4326 in patients with ADPKD (ClinicalTrials.gov identifier NCT04536688).

#### 4.1.2. miR-21

miR-21 as an evolutionarily conserved oncogenic miRNA is expressed in many organs, such as heart, lung, and kidney [62]. miR-21 is activated in solid and hematological malignancies, which promotes the tumorigenesis by regulating cancer cell proliferation and apoptosis [63]. miR-21 has also been reported to inhibit apoptosis, and promote inflammation and fibrosis in kidneys [29]. Studies have revealed similarities of aberrant signaling pathways and pathological derangements between tumor and PKD [64]. miR-21 is also upregulated in cystic kidneys from *Pkd1* conditional knockout mice (*Pkd1^flox/flox^:Pkhd1-Cre* mice), *Pkd2* conditional knockout mice (*Pkd2^flox/flox^:Pkhd1-Cre* mice), and HNF1B conditional knockout mice (*Hnf-1β^flox/flox^:Pkhd1-Cre* mice), and in human ADPKD kidneys [36]. miR-21 can be activated by cAMP/CREB pathway, a well-known driver of cyst growth, whereas it is commonly regulated by TGF-β/SMAD pathway in cancer and fibrosis [36,65,66]. Deletion of miR-21 attenuated cyst growth in ADPKD mouse model through inducing cyst epithelial cell apoptosis [36]. Our previous study reported that Smac-mimetic reduced cyst growth in *Pkd1* conditional knockout mice (*Pkd1^flox/flox^:Pkhd1-Cre* mice) by inducing the cyst-lining epithelial cell apoptosis only, which provides the first evidence that induction of cystic epithelial cell apoptosis is a therapeutic strategy in PKD [67]. Several inhibitors of apoptosis (IAPs) expressed in kidneys, including Jag1, Pten, Spry, and Cdc25a, are validated to be targets of miR-21 [68]. In addition, a tumor suppressor gene, programmed cell death 4 (Pdcd4), was also identified as a new target of miR-21 in PKD [36]. Deletion of Pdcd4 develops kidney cysts spontaneously in Pdcd4^−/−^ mice [69]. Thus, the dysregulated expression of miR-21 promotes the cyst growth via cAMP/CREB-miR-21-PDCD4 signaling axis, and has become a novel drug target in PKD. The anti-miR-21 drug, RG-012, has been tested in the Phase 2 clinical trial for the treatment of Alport syndrome, as discussed in detail in the section below, which should guide its application in ADPKD treatment in the future.

#### 4.1.3. miR-199a-5p

It has been reported that 30 miRNAs are differentially regulated in cystic kidneys of Han:SPRD-cy rats by a microarray-based approach [70]. The other study further identified eight miRNAs (miR-199a-5p, -214, -146b, -21, -34a, -132, -31 and -503) that are upregulated in this PKD rat model [71]. miR-199a-5p regulates cancer cell proliferation, fibrosis, cardiac hypertrophy, and angiogenesis, and has been found to be upregulated in ADPKD tissues [37]. miR-199a-5p repressed the expression of CDKN1C/p57, which is a potent tight-binding inhibitor of several G1 cyclin/Cdk complexes and a negative regulator of cell proliferation [72]. Inhibition of miR-199a-5p decreased cell proliferation and increased apoptosis of cyst epithelial cells through targeting CDKN1C/p57 [37].

#### 4.1.4. miR-200

MiRNAs that may target the differentially expressed mRNAs are predicted by using computational approaches, and nine miRNAs, including miRs-10a, -30a-5p, -96, -126-5p, -182, -200a, -204, -429, and -488, are dysregulated in the kidneys from embryonic PKD1^−/−^ mice [73]. The expression of miR-200 is downregulated in Dicer conditional knockout mice which have deletion of Dicer in mature renal tubules and develop tubular and glomerular cysts [74]. MiR-200 represses the expression of *PKD1* in a posttranscriptional manner in renal epithelial cells via binding with the 3′-UTR of *PKD1* mRNA. Inhibition of miR-200 in renal epithelial cells increases the expression of *PKD1*. It has been reported that *Pkd1* transgenic mice reproducibly develop tubular and glomerular cysts [38]. Thus, the cyst development in Dicer knockout mice may result from the modulation of *PKD1* gene via miR-200.

#### 4.1.5. miR-25-3p

Autophagic influx is inhibited in *Pkd1* knockout mice (*Pkd1^−/−^*), Han:SPRD Cy/Cy rats and congenital polycystic kidney (*cpk*) mice, and induction of autophagy has been found to suppress cyst growth [75]. miR-25-3p is aberrantly expressed in cancers, which regulates cancer cell proliferation and autophagy [39]. miR-25-3p is also upregulated in the cystic kidneys of *Pkd1^flox/-^:Ksp-Cre* mice. Inhibition of miR-25-3p in this *Pkd1* mouse model increased autophagy but decreased renal cell proliferation [39]. miR-25-3p suppressed the autophagy of PKD cells through targeting ATG14, a key player in controlling an autophagy-dependent phosphorylation of beclin-1 [39]. These results suggested that in addition to apoptosis, induction of another regulated cell death, autophagy, via miRNA might also delay cyst growth in ADPKD.

#### 4.1.6. miR-214

Interstitial inflammation and fibrosis caused by accumulation of inflammatory cells is one of the major pathological features of PKD [76]. miR-214 is derived from an lncRNA, dynamin 3 opposite strand (DNM3OS). miR-214 has been reported to play a critical role in remodeling the tumor microenvironment through regulating inflammatory signaling pathways [77]. Both miR-214 and DNM3OS are upregulated in cystic kidneys from *Pkd1* and *Pkd2* mouse models, and human ADPKD, specifically in interstitial cells in the cyst microenvironment [40]. However, deletion of miR-214 in *Pkd1* or *Pkd2* mouse model aggravates the cyst growth. The increased expression of proinflammatory TLR4 and accumulation of pericystic macrophages are observed in the PKD mouse models with miR-214 deletion [40]. TLR4/IFN-γ/STAT1 transcriptionally activates the expression of DNM3OS, the miR-214 host gene. On the other hand, miR-214 directly targets TLR4 and represses its impression to form a negative feedback loop [40]. This study suggested that upregulation of miR-214 in cyst microenvironment has a compensatory protective effect on inhibiting cyst growth and interstitial inflammation.

#### 4.1.7. miR-192, miR-194, and miR-30

Epithelial–mesenchymal transition (EMT) is a process by which epithelial cells transform into mesenchymal cells, which contributes to renal fibrosis in chronic kidney disease [78]. EMT has been reported to be associated with cyst expansion in PKD [79]. By genome-wide analyses of miRNA expression and DNA methylation status in end-stage ADPKD, miR-192 and miR-194 were found to be downregulated due to hypermethylation [41]. The downregulated miR-192 and miR-194 were found to contribute to EMT through direct derepression of ZEB2 and CDH2 in ADPKD [41]. Treatment with precursors of miR-192 and miR-194 slowed cyst growth in *Pkd1^fox/flox^:Aqp2-Cre* mice [41]. Further, Magayr et al. identified two kidney-enriched candidate miRNA families (miR-192/miR-194 and miR-30) through profiling human urinary exosome miRNA by global small RNA-sequencing from early and late stage of ADPKD [42]. Five miRNAs from these two families, including miR-192-5p, miR-194-5p, miR-30a-5p, miR-30d-5p and miR-30e-5p, are validated to be downregulated in ADPKD patient urine exosomes, and cystic kidneys from *Pkd1* mutant mice and ADPKD patients [42]. The growth factors/receptor tyrosine kinases (RTKs), Notch, Wnt/β-catenin, and TGF-β signaling pathways are predicted to be affected by these downregulated miRNAs. PIK3R1 and ANO1 are identified to be novel targets of miR-194-5p, which are increased in ADPKD and promote cyst growth. Additionally, this subset of urinary exosomal miRNAs could serve as novel biomarkers for disease progression as all five miRNAs showed significant correlations with baseline eGFR and ultrasound-determined mean kidney length [42].

#### 4.1.8. miR-193b-3p

By comparing the miRNA profile in human normal and ADPKD cells, five miRNAs were differentially expressed by more than twofold in ADPKD cells [43]. Among them, miR-193b-3p, a tumor suppressor, is downregulated in human ADPKD cells, which results in the increase in the expression of its target, EGF/ErbB family receptor ErbB4 [43,80]. Ligand-induced activation of ErbB4 promotes cyst expansion by driving cystic cell proliferation in ADPKD [43].

#### 4.1.9. miR-501-5p

The roles of p53 and mTOR signaling pathways in PKD have been widely studied [7,81,82]. miR-501-5p is upregulated in ADPKD cells and tissues, which regulates cyst growth by p53 and mTOR signaling pathways [44]. miR-501-5p represses the expression of PTEN and TSC1, leading to the activation of mTOR kinase [44]. The activated mTOR signaling promotes p53 ubiquitination medicated by MDM2 [44]. Thus, inhibition of miR-501-5p decreases cell proliferation and induces apoptosis by inactivation of mTOR and restoring of p53 function in ADPKD cells.

#### 4.1.10. miR-182-5p, miR-20b-5p and miR-106a-5p

miR-182-5p has been identified as one of 13 differentially expressed miRNAs in the kidneys from *Pkd1^flox/flox^:HoxB7-Cre* mouse that have a condition deletion of *Pkd1* in the collecting ducts [45]. The upregulated miR-182-5p regulates actin cytoskeleton rearrangement by repressing its target genes, Wasf2, Dock1, and Itga4, suggesting that miR-182-5p-mediated defects of actin cytoskeleton promote cyst progression [45]. Our recent study found that p68, an RNA helicase, promoted the expression and maturation of miR-17, miR-200c, and miR-182-5p, and further inhibited the expression of *Pkd1* [83]. It has also been reported that miR-20b-5p and miR-106a-5p are downregulated in kidneys from *Pkd2^flox/flox^:HoxB7-Cre* mice that have a conditional deletion of *Pkd2* in the collecting duct [46]. The decreased miR-20b-5p and miR-106a-5p leads to the upregulation of their target, Kruppel-like factor (Klf12), which promotes cyst growth by increasing cell proliferation [46].

#### 4.1.11. LnRNAs in ADPKD

The dysregulated lncRNAs in *Pkd1^flox/flox^:Ksp-Cre* mice and *Pkd2^flox/flox^:Pkhd1-Cre* mice are identified by deep RNA-seq, in which 139 lncRNAs are dysregulated in *Pkd1* conditional knockout kidneys and 106 lncRNAs are dysregulated in *Pkd2* conditional knockout kidneys [84]. The expression of 50 unique lncRNAs changes more than twofold in both *Pkd1* and *Pkd2* mouse models. Among the most dysregulated lncRNAs, Hoxb3os is evolutionarily conserved and highly expressed in the kidney tissues, which is downregulated in kidneys from *Pkd1* and *Pkd2* conditional knockout mice. The expression of human ortholog HOXB3-AS1 is decreased in kidneys from ADPKD patients. Knockout of Hoxb3os in mIMCD3 cells by CRISPR/Cas9 activates the phosphorylation of mTOR and its downstream targets, including p70 S6 kinase, ribosomal protein S6, and the translation repressor 4E-BP1. The mIMCD3 cells with Hoxb3os deletion have increased mitochondrial respiration, which is consistent with the activation of mTOR signaling pathway [84]. These findings suggest that downregulation of Hoxb3os may be through the activation of mTOR and the metabolism of mitochondrial to promote cyst growth in ADPKD.

### 4.2. miRNAs in Autosomal Recessive Polycystic Kidney Disease (ARPKD)

ARPKD is caused by mutations of the PKHD1 gene and is characterized by enlarged kidneys and congenital hepatic fibrosis [85]. It has been reported that miRNAs also contribute to the pathogenesis of ARPKD. First, the expression of PKHD1 is post-transcriptionally negatively regulated by miR-365-1 [86]. Second, the downregulation of epithelial sodium channel (ENaC) mediated by miR-9a-5p, which was upregulated in collecting duct cells, contributed to delay cyst growth in salt deficient diet fed PCK rat, an ARPKD model [47].

The role of miRNA in polycystic liver diseases (PLDs) has also been investigated in the PCK rat. The expression levels of miR-15a are downregulated in cholangiocyte cell line PCK-CCL and cystic liver tissues from PCK rats, and in patients with a PLD [48]. Downregulation of miR-15a accelerated cholangiocyte cell proliferation and promoted liver cyst growth through upregulation of its target, the cell-cycle regulator cell division cycle 25A (Cdc25a) [48].

## 5. miRNAs in HNF1β-Associated Kidney Disease

HNF1β is a DNA-binding transcription factor that regulates the expression of genes involved in membrane transport, cell differentiation, and metabolism in renal tubular epithelial cells [87]. Mutations in HNF1B in humans lead to maturity-onset diabetes of the young, type 5 (MODY5), cystic kidney disease, multicystic dysplastic kidneys, glomerulocystic kidney disease, autosomal dominant tubulointerstitial kidney disease, and congenital anomalies of the kidney and urinary tract (CAKUT) [56]. HNF1β is expressed in nephrons and the branching ureteric bud during kidney development. HNF1β is expressed persistently in renal tubular epithelial cells, but not in glomeruli or interstitium of the mature kidney. As an essential transcriptional regulator, HNF1β is required for multiple steps of kidney development, including ureteric bud branching, initiation of nephrogenesis, and nephron segmentation [87]. Conditional knockout of Hnf1b in mouse kidney results in kidney cyst development and renal failure. HNF1β regulates the transcription of multiple cystic disease genes, including *PKD2*, *PKHD1*, *UMOD*, and *GLIS2* [87]. UMOD is associated with medullary cystic kidney disease, and GLIS2 is associated with nephronophthisis. For a direct role in the transcription of *PKD2* and *PKHD1*, HNF1β has been recognized as a modifier in PKD, although mutations in HNF1B do not cause typical polycystic kidney disease [56]. Mutations of *HNF1B* also cause electrolytes disturbances, including hypomagnesemia and hypokalemia. HNF1β regulates the iron transport in kidney through affecting the expression of solute transporters along the nephron. HNF1β also transcriptionally regulated the expression of FXYD2 that encodes the γ subunit of Na+-K+-ATPase. Mutations of FXYD2 lead to hypomagnesemia. It has been proposed that decreased intracellular magnesium concentration causes urinary potassium wasting through the release of the inhibition of renal outer medullary K+ channel (ROMK) [88]. Furthermore, HNF1β can directly regulate the transcription of *UMOD*, *SCL12A1* and *KCNJ10*, which encode uromodulin, Na+-K+-Cl− transporter (NKCC2), and K+ channel Kir5.1, respectively, and are involved in renal potassium handling [87].

**Figure 3:**
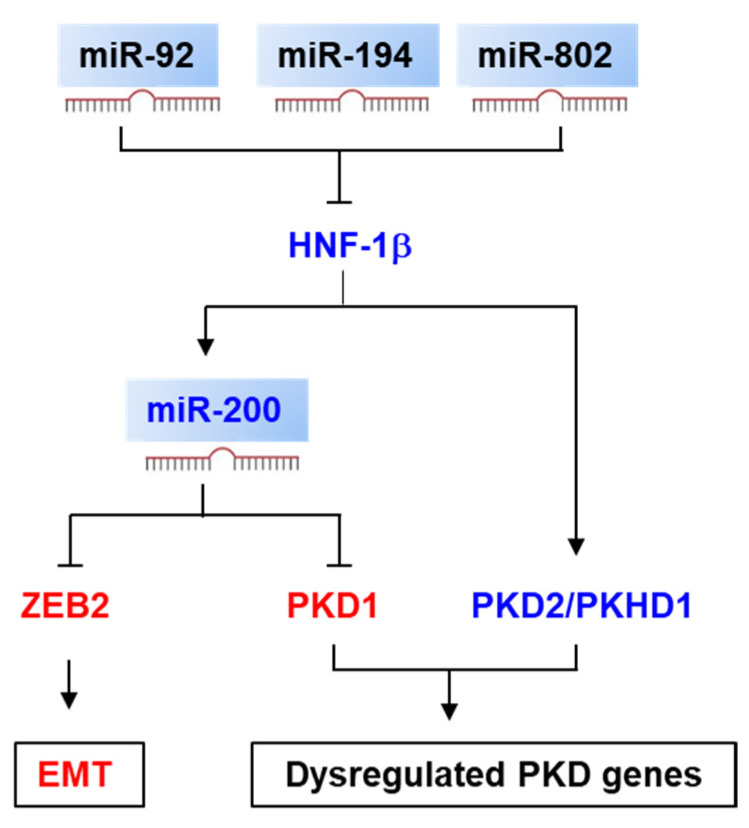
Signaling pathways modulated by miRNAs in HNF1β-associated kidney diseases. miR-92, miR-194 and miR-802 decrease the expression of HNF1β through directly binding with the 3′-UTR of HNF1b, and HNF1β positively regulates the expression of miR-200. Deletion of HNF1β results in the upregulation of ZEB2, a target of miR-200, which promotes the EMT. Deletion of HNF1β also increases the expression of PKD1 via miR-200. In addition, HNF1β directly regulates the transcription of PKD2 and PKHD1. Arrow indicates a positive effect. “T” indicates a negative effect.

The miRNAs that are directly regulated by HNF1β in renal epithelial cells have been identified by ChIP-seq with microarray analysis [89]. The miR-200 family, including miR-200b/200a/429, is a transcriptional target of HNF1β in the renal epithelial cells. Knockout of HNF1β decreased the expression of miR-200 and increased the expression of miR-200 targets, including Zeb2 and Pkd1 in HNF1β knockout mouse kidneys, supporting that HNF-1β regulates EMT and cystic kidney disease via repressing the expression of miR-200 (Figure 3). In addition, mutations of HNF-1β decreased the serum levels of four miRNAs, including miR-24, miR-27b, miR-223 and miR-199a, in MODY5 patients compared to all other diabetes patients and health individuals in the Polish cohort [90]. HNF-1β, on the other hand, can be post-transcriptionally regulated by miRNAs in renal and hepatic cells (Figure 3). It has been found that HNF-1β is negatively regulated by miR-92 in cystic renal epithelial cells [33]. HNF-1β is repressed by miR-194 via binding on a conserved miR-194 binding site located in the 3′-UTR of HNF-1β, leading to decreased cell proliferation and promoting cell apoptosis and migration of mouse metanephric mesenchyme (MM) cells [91]. The 3′-UTR of HNF-1β also has a conserved miR-802 binding site. The expression of miR-802 is increased in the liver of obese mouse models and human individuals with obesity, which could reduce the expression of HNF-1β in the liver, resulting in glucose intolerance, impaired insulin signaling and increased hepatic gluconeogenesis [92].

## 6. miRNAs in Alport Syndrome

Alport syndrome is monogenetic disorder characterized by progressive glomerulonephritis leading to end-stage renal disease at young adult age, ocular anomalies, and hearing defects. Alport syndrome is caused by mutations of genes encoding α3, α4, α5, or α6 chains of collagen type IV, which results in abnormal capillary basement membranes in the kidneys, eyes, and inner ear [93]. The mature mammalian glomerular basement membrane (GBM) contains a subendothelial network and a subepithelial network of type IV collagen. The subendothelial GBM is comprised of collagen type IV α1/α2 heterotrimers. The subepithelial GBM is comprised of collagen type IV α3/α4/α5 heterotrimers and all three chains are required for assembly of these heterotrimers. Mutations of genes encoding α3, α4, α5 chains of collagen type IV result in a thinner GBM [94]. In Alport syndrome, mutant GBM is more susceptible to proteolytic injury than wild type GBM, which leads to the activation of adhesion kinase in podocytes and endothelin A receptors in mesangial cells, and glomerular inflammation, following by progressive tubulointerstitial fibrosis and end-stage renal disease [94].

The expression of miR-21 is higher in tubulointerstitium compared to that in glomeruli of kidneys from a wild type mouse [95]. However, the miR-21 level in the glomeruli is upregulated in the kidneys of Col4α3^−/−^ mice, a murine model of Alport syndrome [95]. The expression of miR-21 in the kidneys of patients with Alport syndrome is significantly increased compared to normal human kidney control [96]. Specifically, miR-21 is highly expressed in the damaged tubular epithelial cells and glomeruli. The elevated levels of miR-21 have a correlation with the disease severity measured by proteinuria, kidney function, and kidney histopathology scores [96].

**Figure 4:**
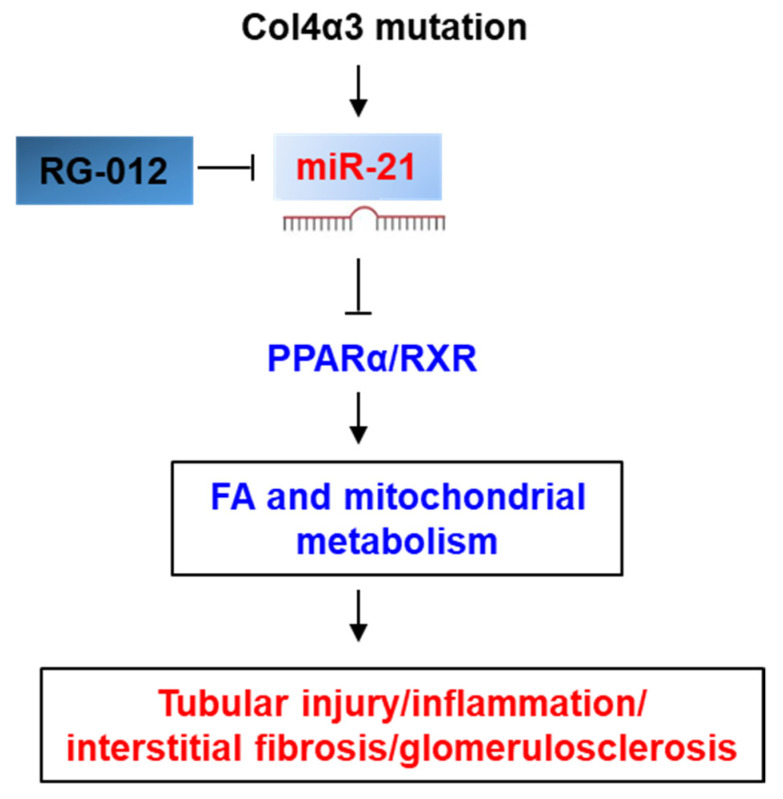
Signaling pathways modulated by miR-21 in Alport syndrome. miR-21 is upregulated in glomeruli and renal tubules in kidneys from patients with Alport syndrome, and in kidneys from *Col4α3^−/−^* mouse model. The upregulation of miR-21 inhibits the expression of PPARα/retinoid X receptor (PPARα/RXR) and its downstream signaling pathways, leading to a dysfunction of fatty acid and mitochondrial metabolism. Treatment with miR-21 antagonism RG-012 improves renal functions by decreasing tubular injury, inflammation, interstitial fibrosis, and glomerulosclerosis in *Col4α3^−/−^* mice. The upregulation or activation of mi-R21 and downstream signaling pathways in Alport syndrome is marked in *red*. The downregulation or inhibition of mi-R21 downstream signaling pathways in Alport syndrome is marked in *blue*. Arrow indicates a positive effect. “T” indicates a negative effect.

The functional role of miR-21 in renal fibrosis has been extensively investigated in various nephropathies [65]. miR-21 contributes to renal fibrogenesis by silencing metabolic pathways, specifically mediated by its targets, PPARα and the mitochondrial inhibitor of reactive oxygen species generation Mpv17l in the unilateral ureteral obstruction model [97]. The anti-miR-21 oligonucleotides are chemically modified single-stranded RNA molecules with full sequence complementarity to miR-21. Treatment with anti-miR-21 oligonucleotides reduced glomerulosclerosis, interstitial fibrosis, tubular injury and inflammation, and therefore improved the survival of Col4α3^−/−^ mice [95]. Targeting of miR-21 activated PPARα/retinoid X receptor (PPARα/RXR) and its downstream signaling pathways in podocytes, tubular, and interstitial cells (Figure 4) [95]. Treatment with anti-miR-21 oligonucleotides also improved mitochondrial function by reducing mitochondrial ROS production [95]. Based on the protective effect against renal fibrosis and inflammation of anti-miR-21 oligonucleotides in Col4α3^−/−^ mouse model, a Phase 2, randomized, double-blind, placebo-controlled clinical trial (ClinicalTrials.gov identifier NCT02855268) to assess the safety, efficacy, pharmacodynamics, and pharmacokinetics of an anti-miR-21 agent RG-012 in patients with Alport syndrome is in progress [98].

In addition, with RNA-seq analysis to profile the expression of miRNAs in the induced pluripotent stem cells (iPSCs) generated from renal tubular cells of patient with Alport syndrome and normal control [99], 155 differentially expressed miRNAs have been identified. Among these miRNAs, hsa-mir-4651, hsa-mir-4461 and hsa-miR-4775 are confirmed to be upregulated in the iPSCs from patients with Alport syndrome [99], suggesting a role of these miRNAs in Alport syndrome.

## 7. miRNAs in Congenital Abnormalities of the Kidney and Urinary Tract (CAKUT)

CAKUT has a wide phenotypic spectrum of developmental defects [100]. The phenotypes of CAKUT consist of renal agenesis and hypodysplasia, cystic kidney disease, dysplastic kidney, hydronephrosis, ureteropelvic junction obstruction, ureter malformations, and vesicoureteral reflux. CAKUT accounts for approximately 50% of pediatric end-stage renal disease [100]. Less than 18% of CAKUT cases are caused by established monogenic mutations [101]. Single-gene mutations from approximately 40 different genes (25 dominant and 15 recessive) have been identified to be causes for CAKUT [101]. The association of miRNAs with the CAKUT-causing genes is not the focus of this review but has been extensively reviewed by Marrone et al. [102]. Many of the CAKUT-causing genes encode transcription factors that play the crucial roles in nephrogenesis, such as HNF1B, PAX2, and FOXC1. MicroRNAs are essential regulators of gene expression through directly binding with mRNA 3′-UTR of targets genes, such as that of miR-92 and miR-194 on HNF1B, suggesting a potential role of microRNAs in CAKUT.

Genetic deletion of microRNA-processing Dicer in developing renal tubules and ureteric buds leads to renal failure and animal death at 4–6 weeks of age [103]. The phenotypes of Dicer knockout mice include small kidney due to decreased number of nephrons, and hydronephrosis due to ureteropelvic junction obstruction. The renal hypoplasia is caused by reduction in tubular branching, and the anomalies of ureters in kidneys result from a defect in the differentiation of ureteric smooth muscle cells in Dicer knockout mice [103]. In addition, conditional knockout of another essential miRNA-processing enzyme (Dgcr8) in the distal nephrons and ureteric buds leads to severe hydronephrosis, kidney cyst, progressive renal failure, and premature death, which resembles the phenotype of Dicer knockout mice [104]. The results suggested an essential role of microRNA-dependent gene regulation in the kidney development. Thus, dysregulation or mutations of miRNAs may be the cause of CAKUT. In addition, seven miRNAs are identified to be associated with CAKUT by integration of microarray gene expression and miRNA target predictions of the ureter samples from pediatric CAKUT patients and control individuals [105]. The expression level of hsa-miR-144 underwent up to a 5.7-fold increase in ureter tissue from CAKUT patients. The functional role of hsa-miR-144 in CAKUT needs to be further investigated. Furthermore, by sequencing 96 stem-loop regions of 73 renal developmental miRNA genes in individuals with non-syndromic CAKUT, two miRNAs (MIR19B1 and MIR99A) were identified with the potentially pathogenic variants in two out of 1213 unrelated individuals [106].

## 8. Noncoding RNA in VHL Disease

VHL disease is an autosomal dominant hereditary syndrome, which is characterized by the development of benign and malignant tumors in the several organ systems [107]. VHL disease is caused by germline mutations of the VHL gene, a tumor suppressor gene. The tumors in VHL disease include retinal hemangioblastomas, pheochromocytomas, and renal cyst and clear-cell renal cell carcinoma (ccRCC). The functional loss of VHL gene, such as somatic VHL mutation and promoter hypermethylation, has been reported in the majority of sporadic ccRCC [108]. VHL associates with a series of proteins to form the E3 ligase enzyme complex that ubiquitinates hypoxia-inducible factor alpha (HIFα) for proteasomal degradation. With the absence of VHL, HIFα accumulates in the cell, and dimerizes with HIFβ to form HIFα/β complex, which transcriptionally actives a series of hypoxia-responsive genes, including vascular endothelial growth factor (VEGF), platelet-derived growth factor (PDGF), and others [108]. The downstream signaling pathways activated by hypoxia-responsive genes play critical roles in the tumorigenesis of ccRCC.

Numerous studies have focused on microRNA expression profiles in sporadic ccRCC and have identified multiple oncogenic and oncosuppressive miRNAs and lncRNAs that regulate the main signaling pathways of ccRCC [109,110,111]. However, few reports have so far studied the role of miRNAs in VHL-associated hereditary ccRCC. By analyzing miRNA and mRNA profiles of VHL-associated hereditary ccRCC, sporadic ccRCC, and normal renal tissue [112], a total of 103 miRNAs had been found to be differentially expressed in ccRCC samples compared to normal renal tissues. Two thirds of miRNAs, including 12 upregulated and 56 downregulated miRNAs, are commonly identified in both hereditary and sporadic ccRCC groups. There are 18 miRNAs that are differentially expressed in VHL-associated ccRCC compared to those in sporadic ccRCC. The expression of miR-210 and miR-155 is upregulated in both VHL-associated and sporadic ccRCC. MiR-210 has been reported to regulate the cellular hypoxic response, cell cycle, mitochondrial oxidative metabolism, and angiogenesis in a variety of cancers [113]. miR-155 has been reported to promote tumor growth by reducing VHL mRNA and HIF1 activity during prolonged hypoxia [114,115]. miR-30c-3p and miR-30a-3p are downregulated in both VHL-associated and sporadic ccRCC, which inhibit cell proliferation and angiogenesis through directly targeting HIF2α in ccRCC [116].

## 9. miRNAs in Fabry Disease

Fabry disease is a rare X-linked hereditary lysosomal storage disorder that is caused by mutations in the GLA gene. GLA gene encodes the lysosomal enzyme α-galactosidase-A (αGalA). The absence or decreased activity of αGalA results in lysosomal accumulation of globotriaosylceramide (Gb3) in many cell types throughout the body. The abnormal Gb3 deposition can affect all cell types in kidneys, including podocytes, epithelial and tubular cells. The renal manifestations of Fabry disease are proteinuria and reduced glomerular filtration rate leading to CKD and ESRD. Early diagnosis and timely initiation of treatment with enzyme replacement therapy (ERT) is beneficial in stabilizing renal function and slowing its decline in Fabry nephropathy. However, the efficacy of ERT in advanced Fabry nephropathy decreases as the renal fibrosis is irreversible. Thus, it is necessary to develop biomarkers for early diagnosis, predicting the disease progression and assessing response to ERT.

Jaurretche et al. examined the expression of urinary miRNAs including miR-21, miR-29, miR-192, miR-200, and miR-433 (the known miRNAs associated with renal fibrosis) in normal individuals and patients with Fabry disease [117]. They found that urinary miR-29 and miR-200 that suppress renal fibrosis are decreased in Fabry disease, whereas the levels of miR-21, miR-192 and miR-433 that promote renal fibrosis have no significant difference. A linear correlation between urinary miR-21 and urinary albumin/creatinine ratio is also observed in Fabry disease patients [117]. In addition, the circulating miRNA profile in Fabry disease has been studied by performing microRNA sequencing of the serum samples, and 10 miRNAs are differentially expressed in the serum of patients with Fabry disease compared to those in normal individuals [118]. The circulating miR199a-5p and miR-126-3p are upregulated in Fabry patients, which can be normalized in Fabry patients after ERT [118]. To further determine whether ERT alters the level of circulating miRNAs in Fabry patients, Xiao et al. performed the microRNA sequencing for the serum samples from Fabry patients with or without ERT [119]. A total of 145 miRNAs are identified to be regulated by ERT. Among those miRNAs, miR-1307-5p, miR-21-5p, miR-152-5p and miR-26a-5p were confirmed to be downregulated in the serum of Fabry patients after ERT in a validation cohort [119]. These studies suggested that urinary and circulating miRNAs might be used as biomarkers to assess the disease progression and the efficacy of ERT in Fabry patients.

## 10. Conclusions and Future Perspectives

Emerging evidence supports that miRNAs and lncRNAs are important mediators in the pathophysiology of kidney diseases. The discovery of aberrantly expressed miRNAs in PKD has defined new molecular mechanisms of cystogenesis and provided a rationale for translating the miRNAs research into the clinical setting. The pharmaceutical and biotech companies are mainly working on two types of products, miRNA mimics and antogomiRs. The stability and delivery to the desired site of action are two major challenges of miRNA-based drugs. Although miRNA-based therapeutics have not yet reached the pharmaceutical breakthrough, a recent clinical evaluation of anti-miR-122 therapy has showed encouraging safety and efficacy in patients with HCV infection [120]. The anti-miR-17 treatment in patients with ADPKD, anti-miR-21 treatment in patients with Alport nephropathy, and miRNA mimics in patients with malignant pleural mesothelioma and non-small cell lung cancer are ongoing. The completion of these clinical trials will further support the significance of miRNA-based therapeutics in human kidneys diseases. Many studies have focused on the lncRNAs in diabetic nephropathy, acute kidney injury, and renal cell carcinoma, while these studies have remained primarily descriptive. The elucidation of the role of lncRNAs in PKD and other genetic kidney diseases is still in its infancy. The development of kidney-specific ncRNAs as therapeutic targets and biomarkers in hereditary kidney disease might be a fascinating area of research.

## Figures and Tables

**Table 1:** Genes involved in major inherited kidney disorders.

Kidney Disorder or Syndrome	Characteristic Signs and Features	Genes and Protein	Involved Kidney Structure
Autosomal dominant polycystic kidney disease, type 1	Polycystic kidneys, liver cysts, brain aneurysms, CKD	PKD1, Polycystin 1	Renal tubules
Autosomal dominant polycystic kidney disease 1, type 2	Polycystic kidneys, CKD	PKD2, Polycystin 2	Renal tubules
Autosomal recessive polycystic kidney disease	Polycystic kidneys, liver fibrosis, CKD	PKHD1, Fibrocystin	Renal tubules
HNF1β-associated kidney disease (autosomal dominant)	Renal cyst, diabetes, CAKUT, and other renal manifestations	HNF1B, hepatocyte nuclear factor-1 beta	Renal tubules
Alport syndrome (X-linked)	Nephritis, SND, CKD	COL4A5, Type IV collagen α5 chain	Basement membrane
Alport Syndrome (autosomal recessive)	Alport syndrome or benign familial hematuria	COL4A3, Type IV collagen α3 chain	Basement membrane
	Nephritis, SND, CKD	COL4A4, Type IV collagen α4 chain	Basement membrane
Alport syndrome with leiomyomatosis (X-linked)	Alport syndrom with leiomyomatosis, CKD	COL4A5 and COL4A6, Type IV collagen α5 and α6 chain	Basement membrane
Congenital abnormalities of the kidney and urinary tract (CAKUT) (autosomal dominant or autosomal recessive)	CAKUT, hypodysplasia, cystic kidney disease, dysplastic kidney, hydronephrosis, ureteropelvic junction obstruction, ureter malformations, vesicoureteral reflux	FOXC1, forkhead transcription factor C1	Renal tubules, podocytes, and basement membrane
		HNF1B, hepatocyte nuclear factor-1 beta	Renal tubules
		PAX2, paired box gene 2	Renal tubules
		Other more than 100 genes	
Von-Lippel-Lindau (VHL) disease (autosomal dominant)	Lindau tumor, retinal angiomatosis, pheochromocytoma, renal tumor	VHL, Tumor suppressor gene g7	Renal tubules
Fabry disease (X linked)	Angiokeratoma, FSGS, adult-onset CKD	GLA, α-galactosidaseA (α-galA)	Renal tubules, interstitium, and glomeruli

CKD, chronic kidney disease; SND, sensorineural deafness; FSGS, focal segmental glomerulosclerosis.

**Table 2:** MicroRNAs that are involved in PKD.

miRNA	Model	Expression	Target	Function	Ref.
miR17–92 cluster	Pkd1 mouse Pkd2 mouse Hnf1B mouse Pkhd1 mouse	upregulated	Pkd1, Pkd2, Hnf1B	Decrease the expression of PKD genes	[33]
miR-17	Pkd1 mouse Pkd2 mouse Human ADPKD	upregulated	Pparα	Regulate mitochondrial metabolism, promote cystic cell proliferation and inflammation	[34,35]
miR-21	Pkd1 mouse Pkd2 mouse Hnf1b mouse Pkhd1 mouse	upregulated	Pdcd4	Inhibit cystic cell apoptosis	[36]
miR-199a-5p	Human ADPKD	upregulated	CDKN1C	Promote cell proliferation and inhibit apoptosis of cystic epithelia	[37]
miR-200	Dicer mouse	downregulated	Pkd1	Increase the expression of Pkd1	[38]
miR-25-3p	Pkd1 mouse	upregulated	Atg14	Suppress autophagy and increase cell proliferation	[39]
miR-214	Pkd1 mouse Pkd2 mouse Human ADPKD	upregulated	TLR4	Promote cyst growth and interstitial inflammation	[40]
miR-192	Human ADPKD	downregulated	ZEB2	Promote epithelial–mesenchymal transition	[41]
miR-194	Human ADPKD	downregulated	CDH2	Promote epithelial–mesenchymal transition	[41]
miR-194	Human ADPKD	downregulated	PIK3R1, ANO1	Promote cyst growth	[42]
miR-193b-3p	Human ADPKD	downregulated	ErbB4	Promote cell proliferation	[43]
miR-501-5p	Human ADPKD	upregulated	PTEN, TSC1	Promote proliferation and inhibit apoptosis of cystic epithelia	[44]
miR-182-5p	Pkd1 mouse	upregulated	Wasf2, Dock1, Itga4	Modulate the actin cytoskeleton	[45]
miR-20b-5p, miR-106a-5p	Pkd2 mouse	downregulated	Klf12	Promote cell proliferation	[46]
miR-9a-5p	PCK rat	downregulated by salt deficient diet	ENaC	Promote cyst growth	[47]
miR-15a	PCK rat	downregulated	Cdc25a	Promote proliferation of cholangiocyte cells	[48]
